# Yeast m^6^A Methylated mRNAs Are Enriched on Translating Ribosomes during Meiosis, and under Rapamycin Treatment

**DOI:** 10.1371/journal.pone.0132090

**Published:** 2015-07-17

**Authors:** Zsuzsanna Bodi, Andrew Bottley, Nathan Archer, Sean T. May, Rupert G. Fray

**Affiliations:** 1 The University of Nottingham, School of Biosciences, Sutton Bonington Campus, Loughborough, LE12 5RD, United Kingdom; 2 The University of Nottingham, NASC, Sutton Bonington Campus, Loughborough, LE12 5RD, United Kingdom; Univ. of Edinburgh, UNITED KINGDOM

## Abstract

Interest in mRNA methylation has exploded in recent years. The sudden interest in a 40 year old discovery was due in part to the finding of *FTO*’s (Fat Mass Obesity) N6-methyl-adenosine (m^6^A) deaminase activity, thus suggesting a link between obesity-associated diseases and the presence of m^6^A in mRNA. Another catalyst of the sudden rise in mRNA methylation research was the release of mRNA methylomes for human, mouse and *Saccharomyces cerevisiae*. However, the molecular function, or functions of this mRNA ‘epimark’ remain to be discovered. There is supportive evidence that m^6^A could be a mark for mRNA degradation due to its binding to YTH domain proteins, and consequently being chaperoned to P bodies. Nonetheless, only a subpopulation of the methylome was found binding to *YTHDF2* in HeLa cells.The model organism *Saccharomyces cerevisiae*, has only one YTH domain protein (*Pho92*, *Mrb1*), which targets *PHO4* transcripts for degradation under phosphate starvation. However, mRNA methylation is only found under meiosis inducing conditions, and *PHO4* transcripts are apparently non-methylated. In this paper we set out to investigate if m^6^A could function alternatively to being a degradation mark in *S*. *cerevisiae*; we also sought to test whether it can be induced under non-standard sporulation conditions. We find a positive association between the presence of m^6^A and message translatability. We also find m^6^A induction following prolonged rapamycin treatment.

## Introduction

Internal methylation of mRNA on the N6 position of adenosine has been known for four decades [1, 2]. During the 1970s and 80s, the PuPuACU methylation consensus sequence was established using biochemical approaches (Schibler et al), and its position was directly mapped in specific transcripts [3, 4, 5]. More recent experiments using antibody pulldown and deep sequencing of m^6^A containing mRNA have confirmed the previously identified consensus and expanded to many thousands the number of identified methylated transcripts [6, 7]. The purification of *MTA70* (*METTL3*) as the methyltransferase component of the methylation complex [8] and the first demonstration of a biological function (meiosis in yeast] associated with the mRNA methylation [9] further advanced the field. *FIP37* as the first interactive partner of the methylase *MTA*, was identified by Zhong, *et al* [10], who also revealed that the m^6^A methylation of mRNA is necessary for *Arabidopsis thaliana* (At) embryonic development. Further biological roles were found in Drosophila, connecting the Notch signalling pathway to mRNA methylation [11]. Since then homologues of *FIP37* have been identified as methylation complex members in yeast (*MUM2*) [12], and in human [*WTAP)* [13], and additional components of the mammalian methylase complex have been identified (*METTL14* and KIAA1429/*Virilizer*) [14, 15]. Further advances in the mRNA methylation field were catalysed by the discovery that *FTO* (Fat Mass Obesity), the most frequently associated gene with obesity, is an mRNA m^6^A adenosine demethylase. This suggested a link between mRNA methylation and human diseases, and consequently assigned a central role for this long known mRNA modification [16]. These findings nearly coincided with the publication of the first human and mouse methylome (epitranscriptome) sequencing datasets [6,7], which was soon followed by that of yeast [17] and subsequently Arabidopsis [18]. A key feature of all of these data sets was a concentration of m^6^A in the 3’ end of transcripts, close to the stop codon. This was consistent with the previous bulk, biochemical assignment of ~90% of methylation to a region within 200 nt of the polyadenylation site of Arabidopsis transcripts [19]. Recently Schwartz *et al*. re-sequenced the human and mouse methylome using more stringent conditions, [15] and eliminated previously published falls positives [6, 7]. In this dataset a distinct *WTAP* independent class of methylated sites near or at the transcription start sites (TSS) of messages was described. These sites are the cap adjacent N6,2’-O-dimethyladenosines (m^6^Am), characterised by Wei, *et al*.[20]. The m^6^Am modification is not found in yeast or plants which have a simpler cap0 structure. However, Kruse *et al*. [21] showed that in mice m^6^Am varies according to tissue and transcript type and they suggested a role in translation based upon the importance of the adenosine N6 position for eIF4e C-terminal interactions [22].

It has recently been suggested that in HeLa cells the main function of m^6^A is to target transcripts for degradation through binding to the *YTHDF2* protein and the subsequent delivery to P bodies [23]. Similar observations about message stability were made in embryonic stem cells, where the presence of m^6^A was suggested to abolish the binding of the human antigen R (*HuR*) in the 3’region of specific messages, facilitating their microRNA driven degradation [24]. Recently, Schwartz *et al*. [15] also suggested that the presence of m^6^A negatively correlates with mammalian transcript half-life. However, in *Arabidopsis* an increase of methylated message stability was suggested [18], and in yeast the strong correlation between half-life and methylation was not observed [17]. Yeast may be somewhat different to metazoans in respect of m^6^A function as, according to our current knowledge, in this organism the presence of mRNA methylation is restricted to a single developmental stage, meiosis. The microRNA driven mechanisms are missing in budding yeast, and there is only one obvious YTH domain protein, *Pho92*, that is associated with phosphate metabolism [25], and recently was characterised as m^6^A binding protein (*Mrb1*) [17]. Currently the main characterised function for *Pho92* (*Mrb1*) protein is the degradation of *PHO4* transcripts in haploid vegetative cells [25] and the *PHO4* transcripts are not in the recently published methylated transcript list [17]. This may either mean that the yeast YTH protein has affinity for non-methylated transcripts, or the *PHO4* transcript may be methylated under non sporulating conditions in an *IME4* independent manner. Since one of the major differences between early meiosis and mitosis is the remodelling of translation [26], in this paper we set out to find out if there is any connection between the presence of m^6^A in transcripts and their representation on ribosomes, hence their translatability. It is currently accepted that mRNA methylation is associated only with meiosis induced by nitrogen and fermentable carbon source starvation in yeast. However, meiosis can be induced by long term rapamycin treatment in late log phase cells [27]. We set out to test if methylation of mRNA could be induced in response to rapamycin treatment under non-starving conditions.

## Results

### Methylation globally is enriched on ribosomes

**Fig 1:**
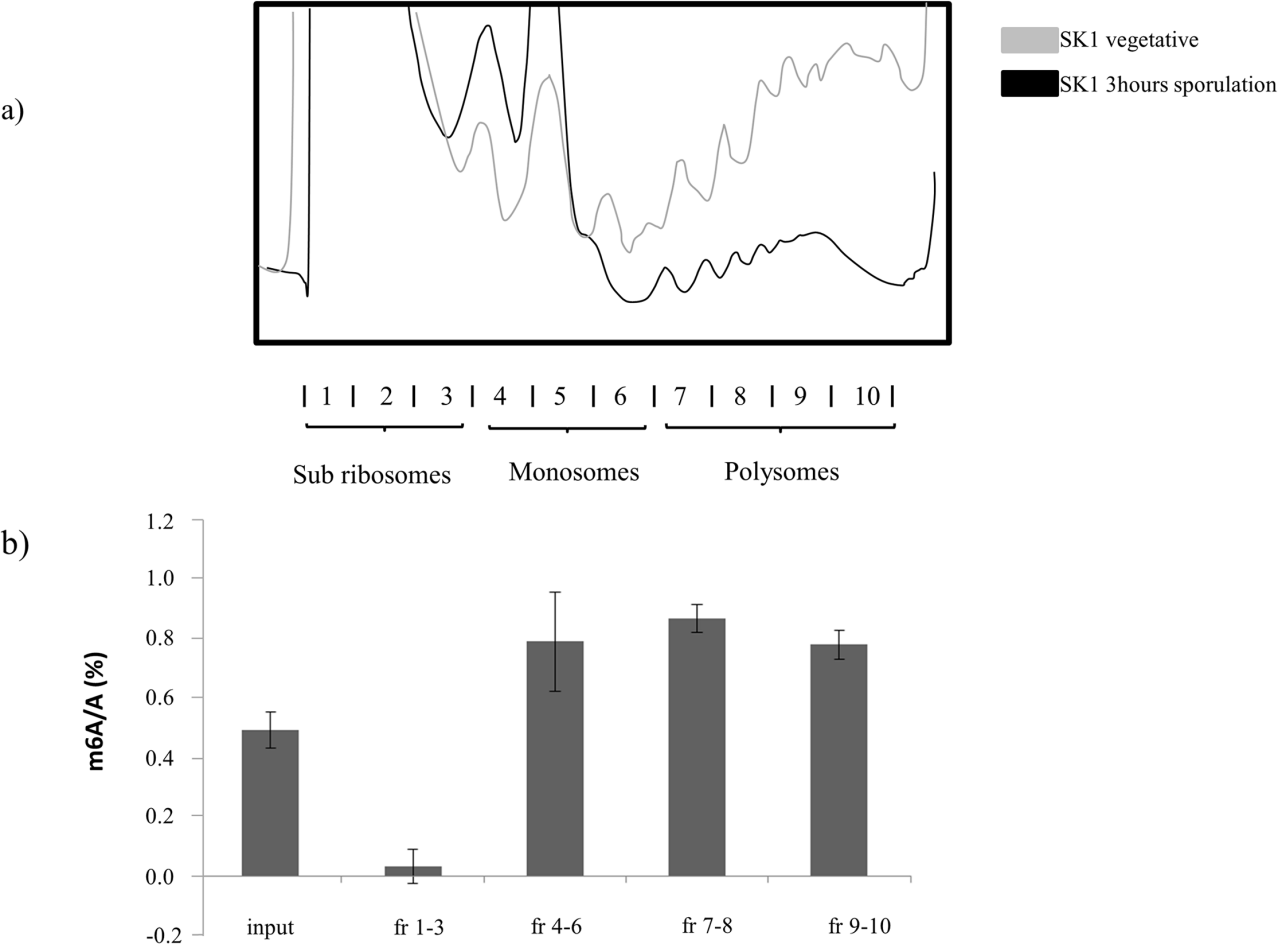
mRNA methylation associated with different polysome fractions. A cell extract from SK1 cells at 3 hours sporulation was fractionated by sucrose gradient centrifugation. The typical polysome profile shows the reduced translation associated with meiosis (a). mRNA extracted from different fractions was radiolabelled and the relative m^6^A content quantified. Input is the start mRNA, before fractionation. All other fractions are pooled fractions; fr1-3 is the subribosomal fractions; fr4-6 is the pooled monosomes; fr7-8 is the lower polysome, and fr9-10 is the higher polysome pooled fraction (Error bar shows SD; each measurement was the average of three biological replicates)(b).

Wang *et al*. [23] showed that in HeLa cells there were only modest differences in the m^6^A to A ratios between subribosomal, monosomal and the polysomal fractions. We set out to test if m^6^A in S. cerevisiae was distributed in a similar way between subribosomes and ribosomes under the specific conditions in which mRNA methylation and meiosis are induced. Meiosis and mRNA methylation were induced in the wt SK1 strain as previously described [28]. After premeiotic synchronisation, the cells were transferred into SPM for 3 hours, at which point cycloheximide was added and after lysis the cells were further processed for polysome fractionation. Fig 1A) shows a typical polysome profile of the SK1 strain 3 hours after the transfer into SPM. The 10 fractions were pooled in 4 groups as follows, group one subribosomal fraction [1–3], followed by monosomal fraction [4–6], lower polysomal fraction [7–8], and higher polysomal, fraction [9–10]. The m^6^A to A ratios were determined using enzymatic digestion and radiolabelling as previously described [10]. The m^6^A to A ratios in the input samples [non-fractionated, starting samples) were also quantified (Fig 1B and S1 Fig). A noticeable enrichment in the mRNA m^6^A content for all pooled ribosomal fractions, compared with input samples was apparent. The subribosomal fractions were depleted for m^6^A in relation to input(Fig 1B and S1 Fig). This shows that in contrast to HeLa cells [23], the cytoplasmic methylated transcripts are almost exclusively associated with ribosomal fractions during early meiosis in yeast.

### Methylation is associated with transcripts enriched on polysome fractions

**Fig 2:**
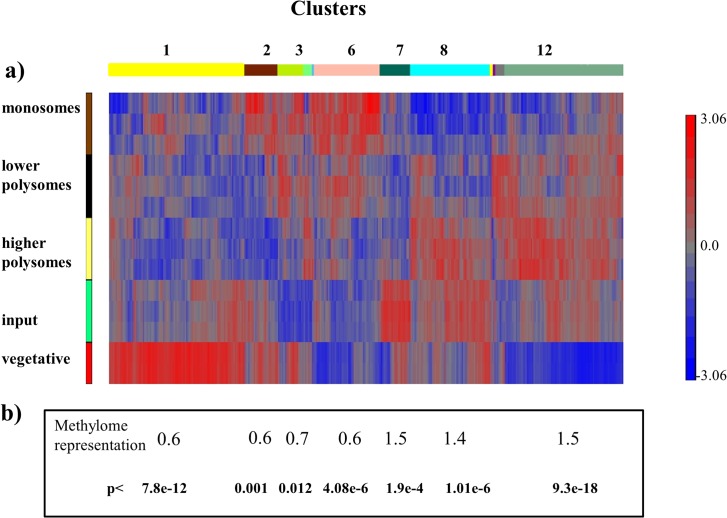
Cluster analysis of transcript polysome occupancy. Total RNA from the different pooled sucrose gradient fractions were extracted and prepared for Affymetrix analysis. The input sample is the non-fractionated total RNA sample. The data for vegetative samples were ‘.cell’ files from Dominissini et al. [6] The raw chip data were analysed using Partek GS 6.0 software package. The gene list of significant genes (FDR<0.05, no fold change cut off was applied) was the base for hierarchic clustering using Partek GS 6.0. 12 different clusters were identified (labelled by different colour bars) (a). The gene lists for each different cluster were used to determine the enrichment for methylated transcripts using the yeast methylome from Schwartz *et al*. [17]. The statistical significance of the overlaps was determined using the online resource http://nemates.org/MA/progs/overlap_stats.html (b).

The total RNA from the pooled ribosomal samples was subjected to microarray analysis to determine the representation of transcripts on the different ribosomal fractions. For this study we used three biological replicates of each: monosomal; lower and higher polysomal fractions; and for the input samples. The total RNA from each sample was processed and hybridized to Affymetrix Gene Chip Yeast Genome 2.0 Arrays. After RMA and ANOVA-1 way analysis, 4094 genes were chosen that were significantly represented (FDR<0.05) in at least one sample type (S1 Table). This list of genes was subjected to hierarchical clustering using Partek Genomics Suite6.0 software. We identified 12 clusters (Fig 2A) and subjected the 7 most populated to further analysis. Cluster 8 and 12 seem to have a lower representation of transcripts on monosomal fractions and higher abundance on the polysomal fractions (Fig 2A) we interpreted this as the indication of more efficient translation. Transcripts in cluster 2 and 6 were mainly represented in the monosome fractions rather than on the polysomes. This indicates a lower translation efficiency of messages associated with these clusters. Cluster 1 did not have any significant enrichment in either the monosomes, or polysomes. This is a cluster with very low level translation.

**Fig 3:**
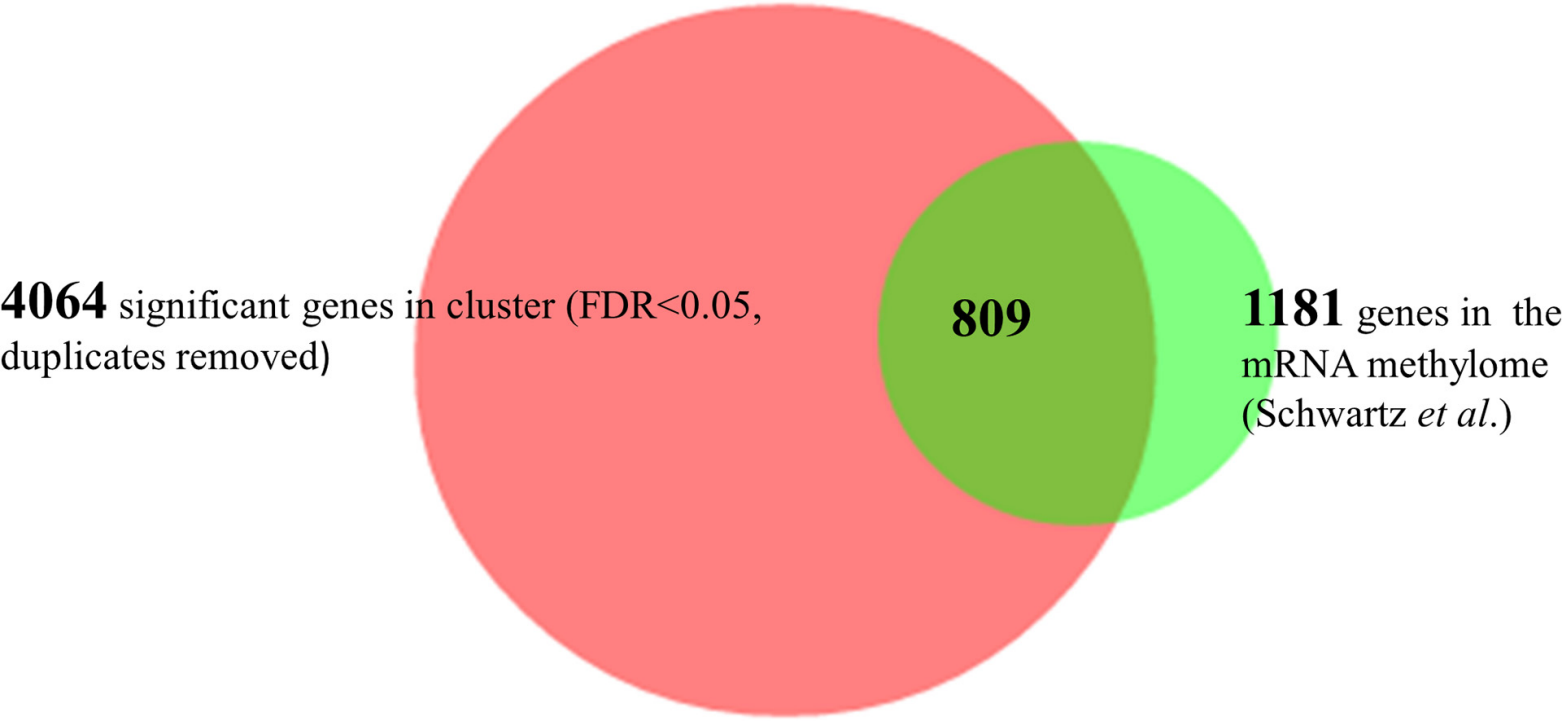
Overlaps between the yeast methylome and the polysome fractions. 809 overlapping genes were identified between the yeast methylome dataset(1181 genes; all duplications were removed) [17] and the genelist for ribosome association (4094 significant genes) used for cluster analysis. The gene list overlaps were determined using the BioVenn web based tool].

We carried out a GO enrichment analysis for the different clusters, using the DAVID annotation online tool. 26% of cluster 1 genes were associated with GOBP translation, 11.5% with GOBP nitrogen compound biosynthetic process, and 9.2% with GOBP mitochondrion organization (S1 Table). This is in agreement with other findings that translation during early meiosis is more restricted than during vegetative life cycle in yeast [26]. The efficiently translating clusters such as, cluster 12 had GO enrichment in GOBP autophagy (9%), GOBP meiosis-1 (6.4%), GOBP cellular response to stress (18%), GOBP protein catabolic processes (12%), GOBP establishment of protein localization (13.4%). (For all of the GO enrichment in the major clusters see S2, S3 and S4 Tables.). The next step in our analysis was to test if there was any enrichment for the methylated transcripts in the clusters where the polysomal representation is high. We identified 809 overlapping genes between the yeast methylome dataset (1181 genes; all duplications were removed) [17] and the genelist (4094 genes) used for cluster analysis (Fig 3).

We looked for enrichment for methylation in individual clusters using the online resource http://nemates.org/MA/progs/overlap_stats.html to determine the statistical significance of the overlaps between the group of genes in different clusters and the mRNA methylome. We found the most significant enrichment of 1.5 fold (p<9.3e^-18^) for methylation in cluster 12, a 1.4 fold enrichment in cluster 8 (p<1.01e^-6^), and 1.5 fold increase (p<1.9e^-4^) in cluster 7 (Fig 2B). A significant depletion for methylation is associated with cluster1(0.6, p<7.8e^-12^), cluster 6 (0.6, p<4.08e^-6^) and cluster 2 (0.6, p<0.001). As *IME2* is a member of cluster 12, we carried out a northern blot to verify the ribosomal distribution of the *IME2* transcripts (S2 Fig). The full length *IME2* transcript nearly exclusively occupied only higher and lower polysomes and not the monosomes or subribosomes. It seems that methylation is mainly enriched in those clusters where translation is most active, and a depletion of methylated transcripts is associated with inefficient translation. However cluster 7 seems to be an exception as in this cluster there is no indication for increased translatability, but there is enrichment for methylated transcripts. This cluster is also enriched for GOBPs associated with transcription (S2 Table).

### Methylation responds to long term rapamycin treatment

We induced sporulation in the SK1 strain using a long term rapamycin treatment [27], a technique that has previously been used for restoring sporulation in industrial yeasts [29]. We grew the SK1 culture in YPD medium to late log phase, at this time rapamycin was added to the culture (final concentration of 200ng/ml), and further incubated.

**Fig 4:**
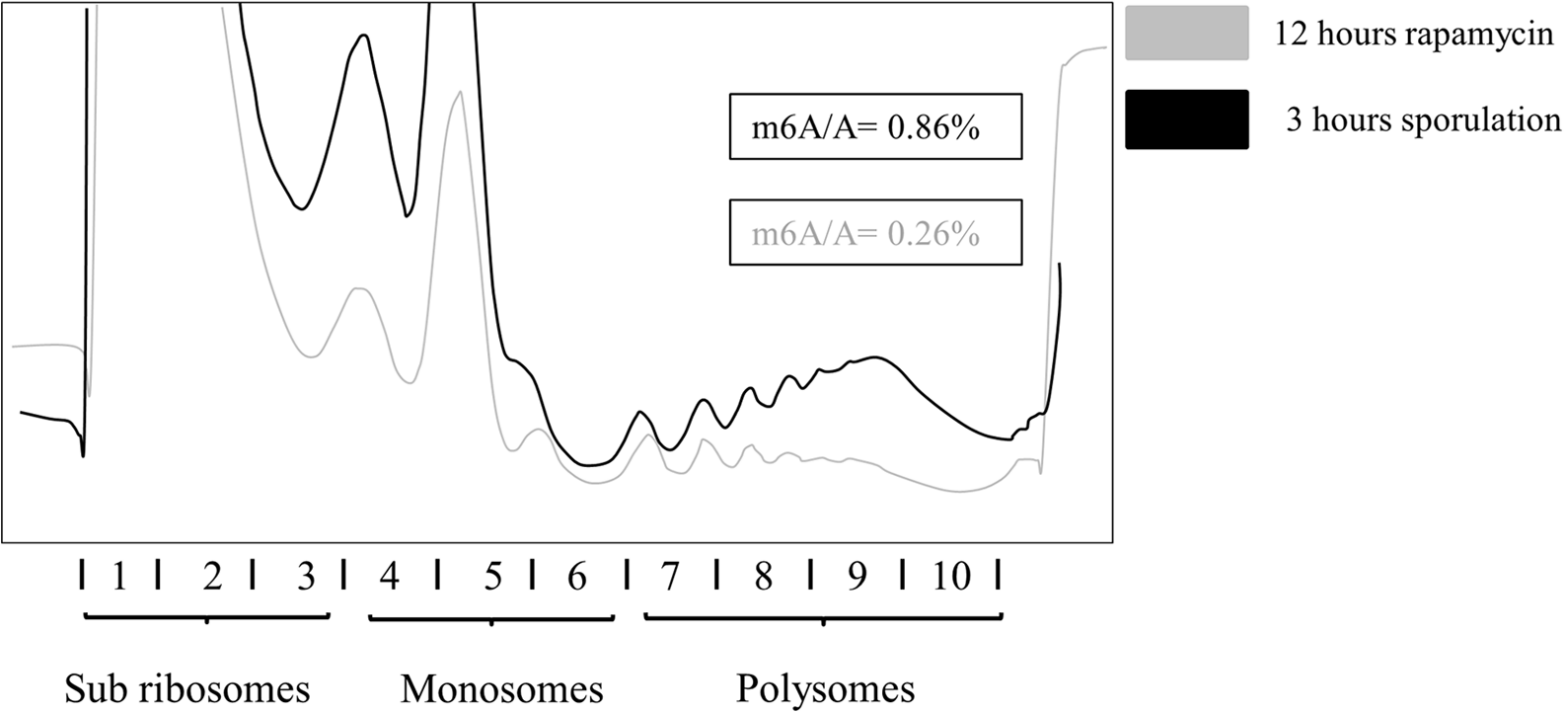
Polysome profiles and methylation levels after rapamycin treatment. After 12 hours rapamycin exposure, (200ng/ml) the cell extract was fractionated using sucrose gradient centrifugation. The m^6^A levels from pooled polysome fraction 7–8 were determined using TLC method (grey colour). Equivalent samples from 3 hour sporulating cells from Fig 1 (black colour) are shown for comparison.

Samples were taken after 2 and 5 hours, at which time no m^6^A was detected in transcripts purified twice using oligo(dT). After 12 hours we were able to detect m^6^A at a 0.08% m^6^A to A ratio. No further increase was observed in methylation levels beyond this point. At this time point no signs of sporulation were observed. This is in accordance with our findings in meiosis that methylation is detected after 3 hours following the induction of sporulation, and it was only later followed by morphological changes [28, 12]. An extract from the 12 hour rapamycin treated cells was subjected to polysome profiling as previously described. A typical profile is represented by Fig 4.

These fractions were pooled in the same way as previously described for the meiotic cultures, and mRNA was isolated, using two rounds of oligo(dT) purification. The m^6^A content of the polysome fractions was quantified using radio labelling and TLC [10]. The m^6^A to A ratios were compared between the polysome fractions and total non-fractionated mRNA samples. A 3-fold increase in methylation levels was detected on the polysomes (m^6^A to A is 0.26%) in comparison to the total mRNA methylation levels (m^6^A to A is 0.08%). These results are consistent with our finding from the 3 hour sporulating samples. Thus, methylated mRNA is associated with translating polysomes irrespective of how meiosis is induced.

### A complete knock out of *IME4* has a basal level of sporulation under long term rapamycin treatment

**Fig 5:**
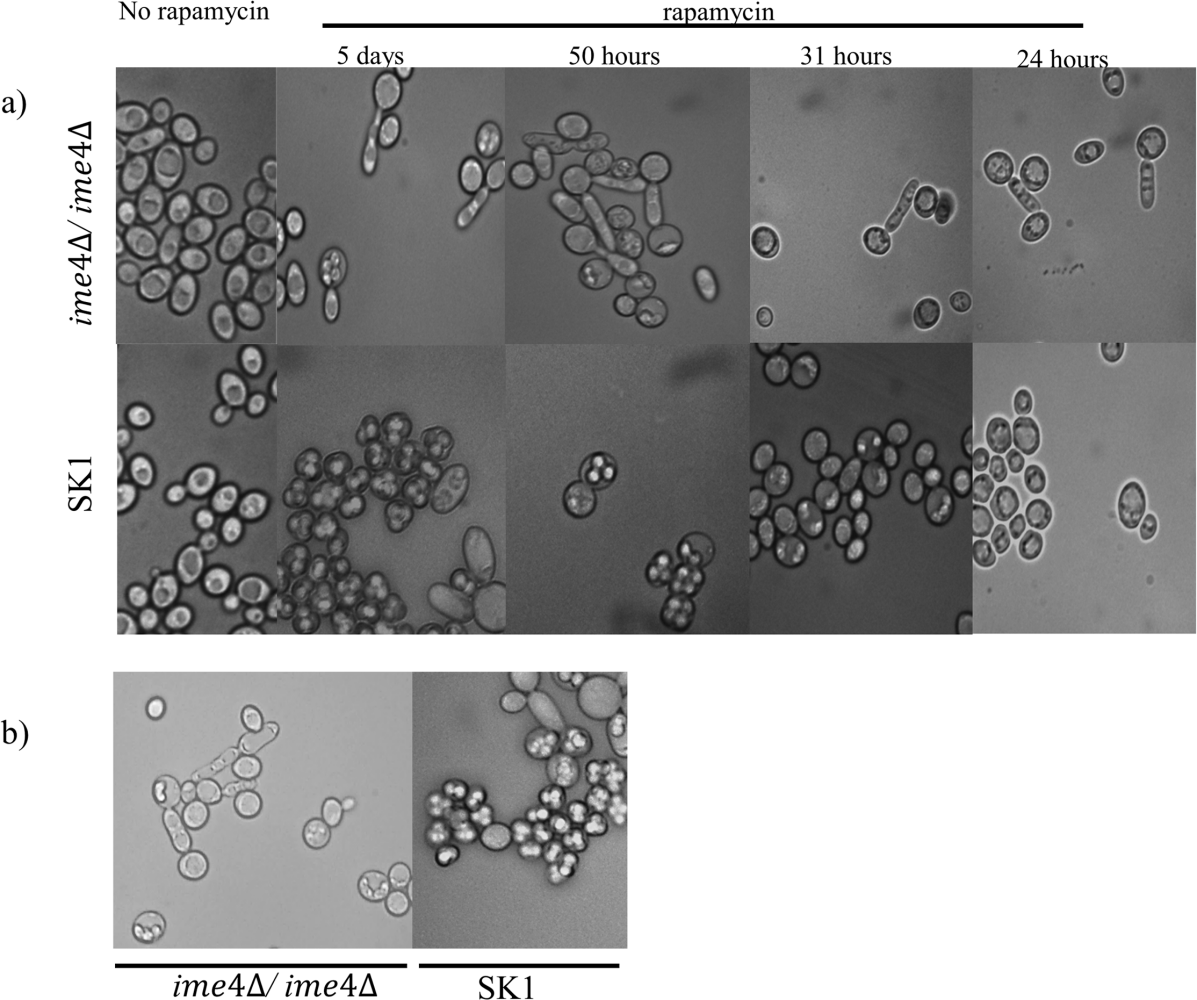
Sporulation phenotype under rapamycin treatment in normal and methylation deficient strains. SK1 and *ime4Δ/ ime4Δ* strains were treated with rapamycin, and at different time points samples were checked for ascus formation. The sporulation pattern of SK1 cells under rapamycin treatment looked similar to sporulation under starvation conditions. Sporulation was completed by the 5^th^ day with a 91±5% sporulation efficiency. The *ime4Δ/ ime4Δ* strain showed some level of sporulation (22.5±12%), but with abnormal ascus formation (a). After 5 days rapamycin treatment the *ime4Δ/ ime4Δ* cells mainly produced elongated cells with only a few producing asci which were all abnormal. In contrast, the SK1 cells maintained a high efficiency of sporulation, although diad and asymmetric asci, with a void volume were common (b).

As previously reported [27] in SK1 cells, sporulation was induced by rapamycin however there were some abnormalities compared to normal starvation-induced meiosis. We treated late log phase cultures of *ime4Δ/ ime4Δ* and SK1 wild type strain with 200ng/ml rapamycin, and samples were monitored for sporulation at different time intervals. We did not observe sporulation before 24 hours. Spore formation become obvious between 24 and 30 hours in the SK1 wt strains, and signs of pseudohyphal-like growths were also observed in the same time period for the *ime4Δ/ ime4Δ* strain (Fig 5A).

In the SK1 wt strains the sporulation was nearly complete (91±5%) within 50 hours after the start of rapamycin treatment. At this point the *ime4Δ/ime4Δ* strains started to show signs of sporulation. However the asci did not appear fully mature (4 spores, and symmetrical), but rather were misshapen, and had a void volume between the ascus and spore wall (22.5±12% ascus) (Fig 5B). The *ime4Δ/ime4Δ* culture rather showed a mixture of elongated, pseudohyphae-like cells and cells with abnormal sporulation pattern (Fig 5B).

## Discussion

Recent reports on the function of mRNA methylation suggest that m^6^A targets transcripts for degradation [23, 24, 15]. This degradation is facilitated by specific binding of the *YTHDF2* protein to m^6^A residues in mRNA, resulting in subsequent delivery of the transcripts to P bodies [23]. This hypothesis is supported by a 14% increase of m^6^A/A level in polysome-derived poly(A) RNA from *YTHDF2* RNAi knock-down cells and a 24% decrease of m^6^A/A in the sub-ribosomal mRNPs. Thus, these authors propose, that binding of *YTHDF2* prevents transcripts from being translated and targets them for degradation (or storage) [23]. The polysome profiles and m^6^A quantification from HeLa cells show that the methylated cytoplasmic transcripts are similarly distributed between cytoplasmic mRNPs and ribosomal fractions (0.33% m^6^A/A in mRNPs, 0.33% in monosomes and 0.47% in polysomes) [23]. This suggests that a significant proportion of methylated transcripts are in mRNP fractions, but not degraded; or that their degradation is a delayed process. Thus, *YTHDF2* may prevent the m^6^A containing mRNA molecules from translation, but does not necessarily target them for quick degradation. In this paper we aimed to test whether similar cytoplasmic distribution can be reproduced for yeast during early meiosis. Therefore we tested the global distribution of m^6^A in the various cytoplasmic mRNP and ribosomal fractions. We also wanted to test whether there is an association between translation and mRNA methylation during meiosis. Translation during meiosis is fundamentally different to that in mitosis—globally there is a net decrease in translation in comparison to vegetative cells that is most apparent in the early stages of meiosis [26]. To investigate this we carried out polysome profiling on early meiotic yeast cultures, and measured the methylation levels on the different pooled polysome fractions. We found a significant enrichment for m^6^A on the ribosomal fractions compared to the non-fractionated starting material. We did not find m^6^A in the subribosomal fractions at these conditions, which contrasts with published results from HeLa cells [23]. This suggests that m^6^A containing messages are not retained as mRNPs during this stage of meiosis.

We determined the transcript populations associated with the different polysomal fractions, using Affymetrix analysis. A hierarchical clustering on the data revealed 12 different clusters in terms of the transcript distribution pattern on different ribosomal fractions. For further analysis we used the most abundant clusters. We compared these data to the yeast methylome data [17] and found significant overlaps of methylated transcripts with those clusters where the mRNA representation was increased on polysome fractions (cl12, cl8); and a significant underrepresentation of methylated species in clusters where messages were mainly associated with monosome fractions (cl6, cl3, cl2). The representation of methylated messages was also decreased in cluster1, where the transcripts were neither associated with monosomes nor polysomes. We have concluded that during early meiosis the most efficiently translated transcripts are enriched in methylation. The only exception to this trend was cluster7 where there was no particular association with either ribosomal fraction. The function of methylation could still be a signal for degradation, but this degradation is likely to be associated with translation. Our data are supportive of an association between translatability and mRNA methylation. We found similar m^6^A enrichment associated with polysome fractions when meiosis was induced using rapamycin. Under this condition global m^6^A levels were lower than during early stages of meiosis [28]. The rapamycin induced meiosis in yeast was different to meiosis under proper starvation conditions; the number of unusual shaped asci is higher in response to rapamycin treatment, and dyad formation is more frequent than during the normal meiotic process [27, 29]. Zheng *et al*. [27] concluded that the proper TOR pathway is necessary in the early stages of meiosis in order to be able to carry out correct spore formation in the later stages of meiosis. When the *ime4Δ/ ime4Δ* strain was treated with rapamycin we observed some sporulation after 5 days of treatment, however, the meiotic products were abnormal, and tetrads with spore number from one to four were observed. None of the asci, even the ones with four spores, were the normal tetrahedral shape. In most cases the spores were squeezed to one end of the ascus which had a void volume between the ascus and spore wall. We would like to suggest that during rapamycin treatment methylation levels are not high enough (m^6^A/A: 0.08%, compared to the meiotic 0.4–0.9%) to regulate the very precise processes needed for symmetrically formed asci with the correct number of spores. Interestingly in the *ime4Δ/ ime4Δ* strain, with undetectable levels of m^6^A, a very low level of sporulation is still possible, induced by rapamycin; however the number of spores per asci and the shape and organization of the asci is somewhat random. Thus the insufficient level of methylation may impede yeast cells from making appropriate developmental choices, which manifests in an abnormal phenotype: the lack of return to symmetrical division in meiosis; less controlled spore numbers; and appearance of apparent pseudohyphal growth.

We conclude that mRNA methylation is enriched in transcripts occupying ribosomal fractions during meiosis induced by starvation and for meiosis induced by rapamycin. This association of m^6^A with ribosomes contrasts yeast to HeLa cells where the most obvious function of m6A is labelling messages for degradation via binding to *YTHDF2*. When the *YTHDF2* is knocked down using RNAi there is an enrichment of m^6^A in transcripts associated with polyribosomes [23]. Thus, unlike human immortalised cells, sporulating yeast m^6^A may promote translation rather than degradation of transcripts.

## Materials and Methods

### Strains and general methods

All analysis was carried out using the SK1 (*can1*), diploid strain (ATCC). Cultures were routinely grown in YPD (1% Bacto-yeast extract, 2% Bacto-peptone, 2% glucose) to the required cell density.

For sporulation experiments, one single colony was inoculated in YPD and grown to 2x10^7^cell/ml. After centrifugation and washing with sterile water, the pellet was resuspended to a 5x10^6^cell/ml density in PSP2 medium [30]. The culture was grown for five generations at 28–30°C with vigorous shaking, and then harvested and washed with water, followed by resuspending in SPM (0.3% potassium acetate, 0.02% raffinose) [30] to a 10^7^cell/ml density to induce sporulation. Following 3 hours vigorous shaking at 28–30°C, the culture was spun down and the pellet was used for RNA extraction, or polysome profiling.

For the rapamycin treatment, SK1 or *ime4Δ/ ime4Δ* cells were grown to a late log phase in YPD and rapamycin was added to a final concentration of 200ng/ml (Cayman Chemical). Following the treatment the cultures were monitored and processed at defined time intervals.

Microscopic images were generated by a Leica DFC320 camera and NIKON OPTIPHOT-2 microscope (400x magnification). Images were processed in the Leica Application Suite 3.3.

### RNA extraction and poly(A) purification

For total RNA extraction we used a hot phenol extraction method [31] for both rapamycin treated and sporulating cultures. Poly(A) RNA was purified using oligo (dT)-Cellulose batch prep following a standard protocol [32]. The oligo(dT) chromatography was carried out twice on each sample and the quality of the mRNA was checked on an RNA 6000 LabChip, with Agilent Bioanalyzer (Ambion).

### m^6^A quantification

The analysis and the quantification of m^6^A in different samples were carried out by applying the method from Zhong *et al*. [10]. For each sample, 50 ng of mRNA was digested with Ribonuclease T1 (1000 units/μL; Fermentas).The fragments were labeled using [γ-^32^P] ATP (6000 Ci/mmol; Perkin-Elmer), followed by digestion with P1 nuclease (Sigma-Aldrich) for 1 hour at 37°C. 1 μl of each sample was loaded on cellulose TLC plates (20 x 20 cm; Merck) and developed in a solvent system of isobutyric acid: 0.5M NH_4_OH (5:3, v/v), as first dimension, and isopropanol: HCl: water (70:15:15, v/v/v), as the second dimension. The quantification of spot intensities, was done using storage phosphor screen (Fuji) and an FX imager in combination with Quantity One 4.6.3. software (Bio-Rad).

### Polysome profiles

The ribosomal complexes in the treated or non-treated yeast cultures were stabilised by adding cycloheximide to a 100μg/ml final concentration prior to harvest. The cell were spun down immediately, and frozen in liquid N_2._ They were either stored at -70°C or processed immediately by grinding in pestle and mortar, under liquid N_2_ to fine powder. The cells were lysed in 0.5ml lysis buffer (0.3M NaCl, 15mM MgCl_2_, 15mM Tris-HCl pH7.5, cycloheximide 100ug/ml, Heparin (sodium salt) 1mg/ml, 1% Triton X-100). The lysates were loaded on a sucrose gradient column and spun for two hours at 38 000 rpm at 4°C. After the gradient centrifugation 12x 1ml fractions were collected and precipitated in equal volume isopropanol. After several washes with 80% ethanol the samples were resuspended in water and processed.

### Microarray analysis

Three biological repeats of 3 hour sporulating yeast cultures were used for polysome profiling. The subribosomal pooled fractions 4, 5 and 6 represented the monosomes. Pooled fractions 7 and 8; and pooled fractions 9 and 10 represented the lower and higher polysomes respectively. RNA was purified from each pooled fraction samples and the non-fractionated cell lysates of each replicate using phenol extraction, followed by ethanol precipitation. The Affymetrix Yeast Genome2.0 array was used for microarray analysis. Total RNA samples were processed and labelled using the GeneChip 3’ IVT Expression Kit, (Affymetrix, cat#901228). Hybridizations were carried out at the NASC’s Affymetrix service (Nottingham Arabidopsis Stock Centre, University of Nottingham, UK), following the standard protocol from the manufacturer (GeneChip Expression Analysis, Affymetrix). The generated ‘.cel’ files for each of the hybridisations are available from GEO (accession ID: GSE68435 http://www.ncbi.nlm.nih.gov/geo/). The raw chip data were analysed using Partek GS 6.0 software. After initial probe-level RMA normalisation the signals were further normalized by standardizing the signal value of each probe-set to the median of that probe-set across all hybridisations. We also imported.cel files for vegetatively growing SK1 cells [6] for the normalization and differential expression. Differentially expressed probe-sets were identified between different polysome fractions and non-fractionated RNA samples of 3 hour sporulating yeast; and between vegetatively growing SK1 cultures and non-fractionated 3 hour sporulating samples. The gene list of significant genes(FDR<0.05, no fold change cut off) was the basis for hierarchical clustering using Partek GS 6.0. The gene list overlaps were determined using the BioVenn web based tool [33]. The GO analysis was done using DAVID online Bioinformatics Resources 6.7 [34, 35].

## Supporting Information

S1 Fig TLC results from different polysome fractions. A crude extract from 3 hours sporulating SK1 cells was subjected to polysome fractionation using sucrose gradient centrifugation as described in the Materials and Methods. The typical polysome profiles for early (3 hours) meiosis and vegetative cycle (a) with the quantification results for m6A to A ratios from different pooled fractions (b) are duplicated from Fig 1 (main text). Example TLCs from one of the 3 repeats are shown (c). The intensities of radio labelled m6A and A spots were measured using a phosphoscreen (Fuji) and the Molecular Imager FX (BIO-RAD) in combination with Quantity One software (BIO-RAD) as previously described [10]. m6A to A ratios were calculated using these values. It should be noted that this method specifically labels nucleotides following a G (as T1 nuclease specifically cuts after G), thus the observed m6A to A ratios from TLC are the ratios of Gpm6A to GpA in the transcripts from which they were derived.
(PDF)

S2 Fig Northern blot showing the *IME2* transcript distribution on different polysome fractions. RNA from different fractions was precipitated using isopropanol and resuspended in equal volumes of water. Equal volumes of RNA samples from each fraction were loaded on a formaldehyde agarose gel (lane 1: ssRNA Ladder, NEB), and after separation the RNA was transferred on to a nylon membrane, and hybridised to a P^32^ labelled *IME2* probe using standard protocols (Primer sequences for IME2 probe: 
For_primer CTATCGCAGATACTGGCTGG;Rev_primer GTAGTAGATCCAACGATGAAC).
(PDF)

S1 Table Gene list used for cluster analysis. (XLSX)

S2 Table GOBP lists. (XLS)

S3 Table GOCC lists. (XLS)

S4 Table GOMF lists. (XLS)

## References

[1] Desrosiers R, Friderici K, Rottman F (1974) Identification of methylated nucleosides in messenger RNA from Novikoff hepatoma cells. Proc. Natl. Acad. Sci. USA, 71, 3971–3975. 4372599 10.1073/pnas.71.10.3971 PMC434308

[2] Perry RP, Kelley DE (1974) Existence of methylated messenger-RNA in mouse L cells. Cell, 1, 37–42.

[3] Nichols JL, Welder L (1981) Nucleotides adjacent to *N*6-methyladenosine in maize poly(A)-containing RNA. Plant Sci. Lett., 21, 75–81.

[4] Horowitz S, Horowitz A, Nilsen TW, Munns TW, Rottman FM (1984) Mapping of *N*6-methyladenosine residues in bovine prolactin mRNA. Proc. Natl. Acad. Sci. USA, 81, 5667–5671. 6592581 10.1073/pnas.81.18.5667 PMC391771

[5] Kane SE, Beemon K (1985) Precise localization of m^6^A in Rous sarcoma virus RNA reveals clustering of methylation sites: Implications for RNA processing. Mol. Cell. Biol., 5, 2298–2306. 3016525 10.1128/mcb.5.9.2298-2306.1985 PMC366956

[6] Dominissini D, Moshitch-Moshkovitz S, Schwartz S, Salmon-Divon M, Ungar L, Osenberg S, et al (2012) Topology of the human and mouse m6A RNA methylomes revealed by m6A-seq. Nature, 485, 201–206 10.1038/nature11112 22575960

[7] Meyer KD, Saletore Y, Zumbo P, Elemento O, Mason CE, Jaffrey SR (2012) Comprehensive analysis of mRNA methylation reveals enrichment in 3′ UTRs and near stop codons. Cell, 149, pp. 1635–1646 10.1016/j.cell.2012.05.003 22608085 PMC3383396

[8] Bokar JA, Shambaugh ME, Polayes D, Matera AG, Rottman FM (1997) Purification and cDNA cloning of the AdoMet-binding subunit of the human mRNA (*N*6-adenosine)-methyltransferase. RNA, 3, 1233–1247. 9409616 PMC1369564

[9] Clancy MJ, Shambaugh ME, Timpte CS, Bokar JA (2002) Induction of sporulation in *Saccharomyces cerevisiae* leads to the formation of *N*6-methyladenosine in mRNA: A potential mechanism for the activity of the *IME4* gene. Nucleic Acids Res., 30, 4509–4518. 12384598 10.1093/nar/gkf573 PMC137137

[10] Zhong S, Hongying L, Bodi Z, Button JD, Vespa L, Herzog M, Fray RG (2008) *MTA* is an Arabidopsis messenger RNA adenosine methylase and interacts with a homolog of a sex-specific splicing factor. The Plant Cell, 20, 1278–1288 10.1105/tpc.108.058883 18505803 PMC2438467

[11] Hongay CF, Orr-Weaver TL (2011) *Drosophila* Inducer of MEiosis 4 (*IME4*) is required for Notch signaling during oogenesis. Proc Natl Acad Sci USA., 108, 14855–60. 10.1073/pnas.1111577108 21873203 PMC3169142

[12] Agarwala S D, Blitzblau HG, Hochwagen A, Fink GR (2012) RNA methylation by the MIS complex regulates a cellfate decision in Yeast. PLoS Genet 8, e1002732 10.1371/journal.pgen.1002732 22685417 PMC3369947

[13] Ping X-L, Sun B-F, Wang L, Xiao W., Yang X., et al (2014) Mammalian WTAP is a regulatory subunit of the RNA N6-methyladenosine methyltransferase. Cell Research, 24,177–189, 10.1038/cr.2014.3 24407421 PMC3915904

[14] Liu J, Yue Y, Han D, Wang X, Fu Y, Zhang L, Jia G, Yu M, Lu Z, Deng X et al (2014) A METTL3- METTL14 complex mediates mammalian nuclear RNA N-adenosine methylation. Nat Chem Biol. 10, 93–95. 10.1038/nchembio.1432 24316715 PMC3911877

[15] Schwartz S, Mumbach MR, Jovanovic M, Wang T, Maciag K, et al (2014) Perturbation of m6A writers reveals two distinct classes of mRNA methylation at internal and 5′ sites. Cell Reports, 8, 284–296, 10.1016/j.celrep.2014.05.048 24981863 PMC4142486

[16] Jia G, Fu Y, Zhao X, Dai Q, Zheng G, Yang Y, et al. (2012) N6-methyladenosine in nuclear RNA is a major substrate of the obesity-associated *FTO* . Nat. Chem. Biol., 7, 885–887 10.1038/nchembio.687 PMC3218240 22002720

[17] Schwartz S, Agarwala SD, Mumbach MR, Jovanovic M, Mertins P, et al. (2013) High-resolution mapping reveals a conserved, widespread, dynamic mRNA methylation program in yeast meiosis. Cell, 155, 1409–1421 10.1016/j.cell.2013.10.047 24269006 PMC3956118

[18] Luo G, MacQueen A, Zheng G, Duan H, Dore LC, et al (2014) Unique features of the m^6^A methylome in *Arabidopsis thaliana* . Nat Commun, 5, 5630, 10.1038/ncomms6630 25430002 PMC4248235

[19] Bodi Z, Zhong S, Mehra S, Song J, Graham N, Li H, May S, Fray RG (2012) Adenosine methylation in Arabidopsis mRNA is associated with the 3′ end and reduced levels cause developmental defects. Fron. Plant Sci. 3, 48 10.3389/fpls.2012.00048 PMC3355605 22639649

[20] Wei C M, Gershowitz A, Moss B (1976) 5’-terminal and internal methylated nucleotide-sequences in Hela-cell messenger-RNA. Biochemistry, 15, 397–401 174715 10.1021/bi00647a024

[21] Kruse S, Zhong S, Bodi Z, Button J, Alcocer MJC, Hayes CJ, et al (2011) A novel synthesis and detection method for cap-associated adenosine modifications in mouse mRNA. Scientific Reports 1, 126, 10.1038/srep00126 22355643 PMC3216607

[22] Tomoo K, Shen X, Okabe K, Nozoe Y, Fukuhara S, Morino S (2002) Crystal structures of 7-methylguanosine 5‘-triphosphate (m(7)GTP)- and P-1-7-methylguanosine-P-3-adenosine-5‘,5‘-triphosphate (m7GpppA)-bound human full-length eukaryotic initiation factor 4E: biological importance of the C-terminal flexible region. Biochem. J., 362, 539–544 11879179 10.1042/0264-6021:3620539 PMC1222416

[23] Wang X, Lu Z, Gomez A, Hon GC, Yue Y, et al (2014) N^6^-methyladenosine-dependent regulation of messenger RNA stability. Nature, 505, 117–120 /nature12730 24284625 10.1038/nature12730 PMC3877715

[24] Wang Y, Li Y, Toth JI, Petroski MD, Zhang Z, Zhao JC (2014) N6-methyladenosine modification destabilizes developmental regulators in embryonic stem cells. Nat Cell Biol, 16, 191–198, 10.1038/ncb2902 24394384 PMC4640932

[25] Kang H, Jeong S, Kim K, Baek I, Chang M, Kang C, et al (2014) A novel protein, Pho92, has a conserved YTH domain and regulates phosphate metabolism by decreasing the mRNA stability of PHO4 in Saccharomyces cerevisiae. Biochem. J., 457, 391–400, 10.1042/BJ20130862 24206186

[26] Brar GA, Yassour M, Friedman N, Regev A, Ingolia NT, Weissman JS (2012) High-Resolution View of the Yeast Meiotic Program Revealed by Ribosome Profiling. Science, 335, 552–557, 10.1126/science.1215110 22194413 PMC3414261

[27] Zheng X-F, Schreiber SL (1997). Target of rapamycin proteins and their kinase activities are required for meiosis. Proc Natl Acad Sci USA., 94, 3070–3075. 9096347 10.1073/pnas.94.7.3070 PMC20323

[28] Bodi Z, Button JD, Grierson D, Fray RG (2010) Yeast targets for mRNA methylation. Nucleic Acids Res., 38, 5327–35. 10.1093/nar/gkq266 20421205 PMC2938207

[29] Nakazawa N, Niijima S, Tanaka Y, Ito T (2012) Immunosuppressive drug rapamycin restores sporulation competence in industrial yeasts. J. Biosci. Bioeng., 113, 491–5, 10.1016/j.jbiosc.2011.11.026 22197499

[30] Kassir Y, Simchen G (1991) Monitoring meiosis and sporulation in *Saccharomyces cerevisiae* In Guthrie C. and Fink G.R.(eds), Methods in Enzymology. Academic Press Inc., San Diego, Vol. 194, pp. 94–110. 2005827 10.1016/0076-6879(91)94009-2

[31] Schmitt ME, Brown TA, Trumpower BL (1990) A rapid and simple method for preparation of RNA from *Saccharomyces cerevisiae* . Nucleic Acids Res., 18, 3091–3092. 2190191 10.1093/nar/18.10.3091 PMC330876

[32] Sambrook J, Russel DW (2001) Molecular Cloning: A Laboratory Manual. Cold Spring Harbor Laboratory Press, Cold Spring Harbor, NY., 7.13–7.17.

[33] Hulsen T, de Vlieg J, Alkema W (2008) BioVenn—a web application for the comparison and visualization of biological lists using area-proportional Venn diagrams.BMC Genomics, 9, 488 10.1186/1471-2164-9-488 18925949 PMC2584113

[34] Huang DW, Sherman BT, Lempicki RA (2009) Systematic and integrative analysis of large gene lists using DAVID Bioinformatics Resources. Nat Protoc., 4, 44–57. 10.1038/nprot.2008.211 19131956

[35] Huang DW, Sherman BT, Lempicki RA (2009) Bioinformatics enrichment tools: paths toward the comprehensive functional analysis of large gene lists. Nucleic Acids Res., 37, 1–13. 10.1093/nar/gkn923 19033363 PMC2615629

