# Supplementary material for: Yeast m6A Methylated mRNAs Are Enriched on Translating Ribosomes during Meiosis, and under Rapamycin Treatment
Source: PLoS One. 2015 Jul 17;10(7):e0132090. doi: 10.1371/journal.pone.0132090 (PMC4505848; doi:10.1371/journal.pone.0132090)
Supplement: S1 Fig — A crude extract from 3 hours sporulating SK1 cells was subjected to polysome fractionation using sucrose gradient centrifugation as described in the Materials and Methods. The typical polysome profiles for early (3 hours) meiosis and vegetative cycle (a) with the quantification results for m6A to A ratios from different pooled fractions (b) are duplicated from Fig 1 (main text). Example TLCs from one of the 3 repeats are shown (c). The intensities of radio labelled m6A and A spots were measured using a phosphoscreen (Fuji) and the Molecular Imager FX (BIO-RAD) in combination with Quantity One software (BIO-RAD) as previously described [10]. m6A to A ratios were calculated using these values. It should be noted that this method specifically labels nucleotides following a G (as T1 nuclease specifically cuts after G), thus the observed m6A to A ratios from TLC are the ratios of Gpm6A to GpA in the transcripts from which they were derived. (PDF) [file pone.0132090.s001.pdf]

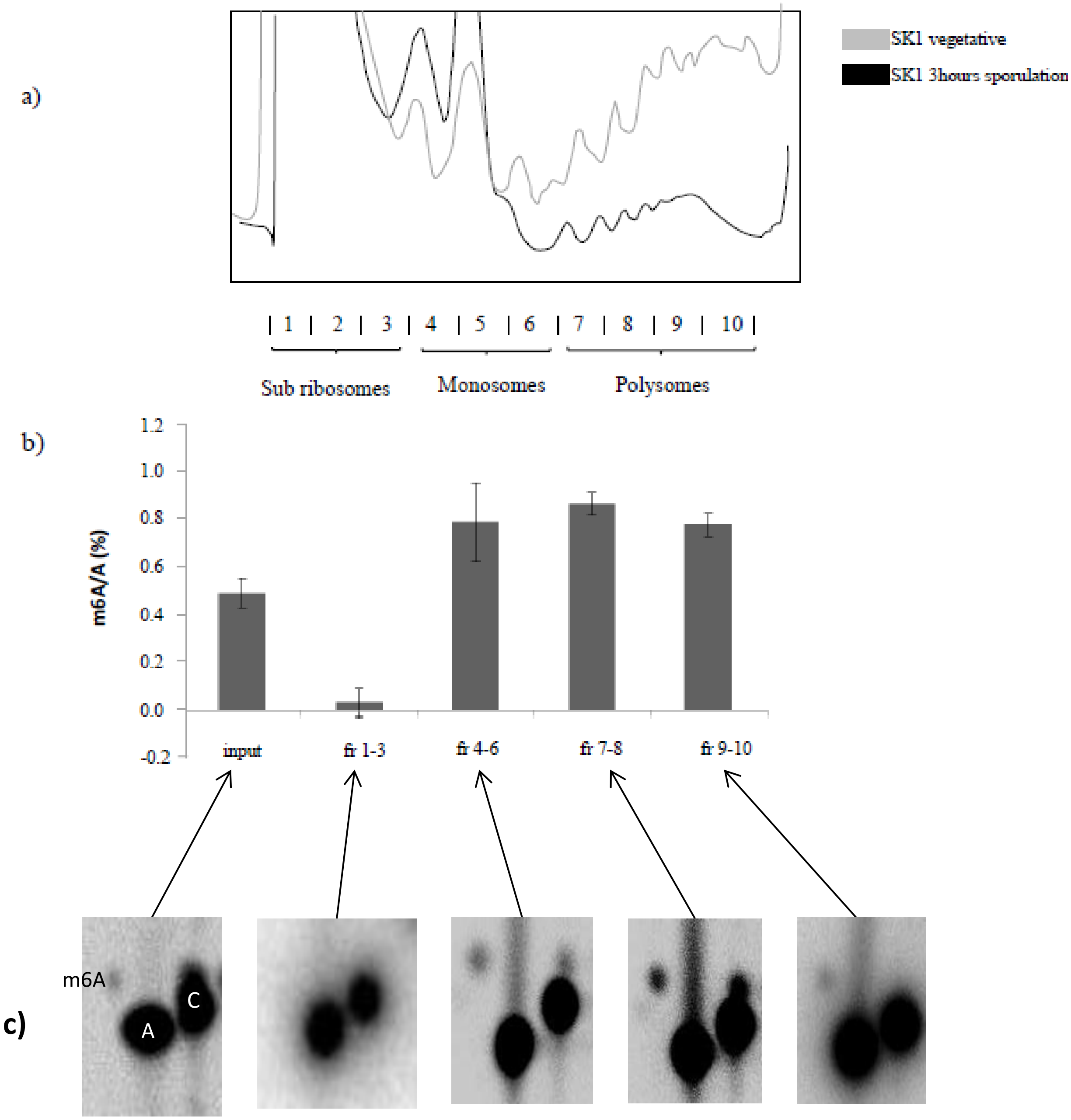

S1 Figure TLC results from different polysome fractions

A crude extract from 3 hours sporulating SK1 cells was subjected to polysome fractionation using sucrose gradient centrifugation as described in the Materials and Methods. The typical polysome profiles for early (3 hours) meiosis and vegetative cycle (a) with the quantification results for m^6A to A ratios from different pooled fractions (b) are duplicated from Fig. 1 (main text).

Example TLCs from one of the 3 repeats are shown (c). The intensities of radio labelled m^6A and A spots were measured using a phosphoscreen (Fuji) and the Molecular Imager FX (BIO-RAD) in combination with Quantity One software (BIO-RAD) as previously described [10]. m^6A to A ratios were calculated using these values. It should be noted that this method specifically labels nucleotides following a G (as T1 nuclease specifically cuts after G), thus the observed m^6A to A ratios from TLC are the ratios of Gpm^6A to GpA in the transcripts from which they were derived.
