# Supplementary material for: Yeast m6A Methylated mRNAs Are Enriched on Translating Ribosomes during Meiosis, and under Rapamycin Treatment
Source: PLoS One. 2015 Jul 17;10(7):e0132090. doi: 10.1371/journal.pone.0132090 (PMC4505848; doi:10.1371/journal.pone.0132090)
Supplement: S2 Fig — RNA from different fractions was precipitated using isopropanol and resuspended in equal volumes of water. Equal volumes of RNA samples from each fraction were loaded on a formaldehyde agarose gel (lane 1: ssRNA Ladder, NEB), and after separation the RNA was transferred on to a nylon membrane, and hybridised to a P32 labelled IME2 probe using standard protocols (Primer sequences for IME2 probe: For_primer CTATCGCAGATACTGGCTGG;Rev_primer GTAGTAGATCCAACGATGAAC). (PDF) [file pone.0132090.s002.pdf]

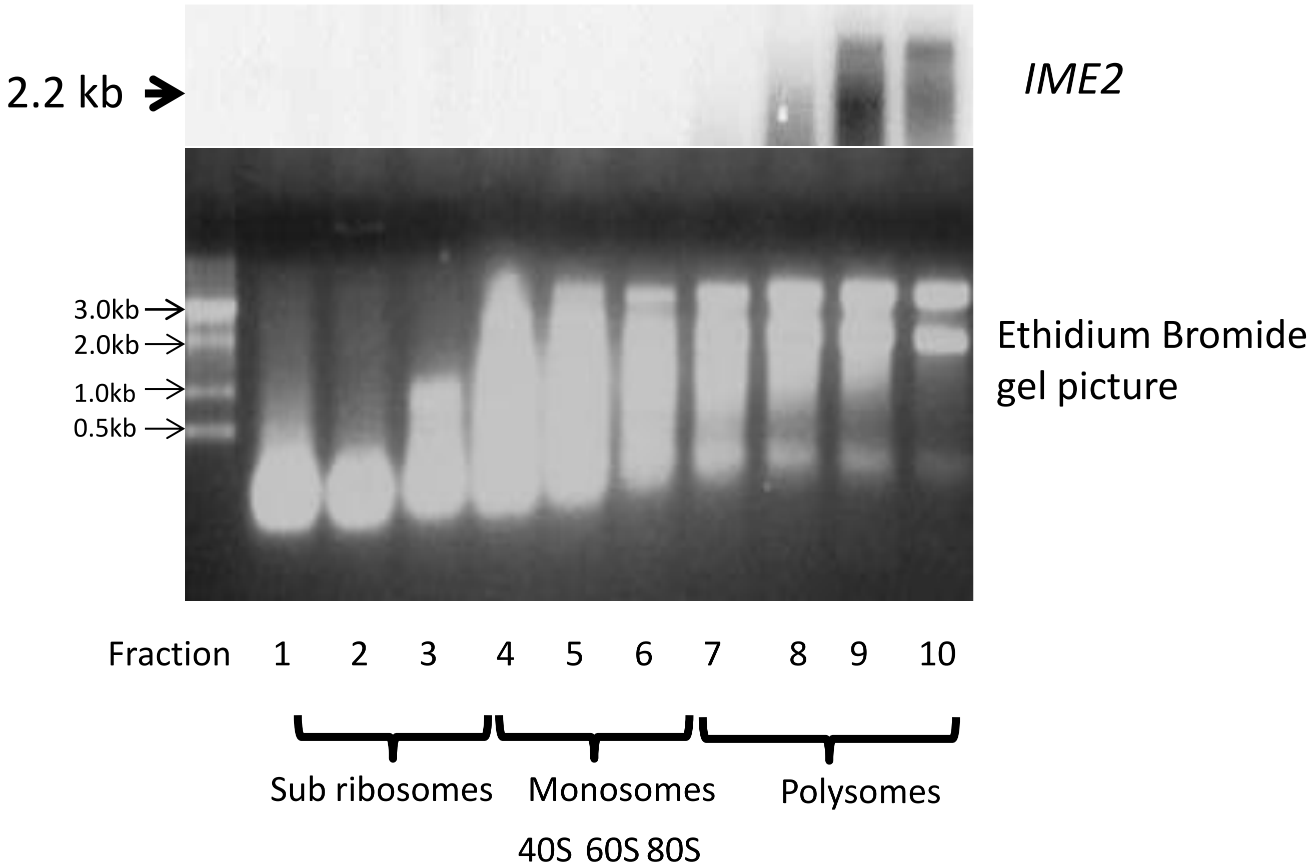

S2 Figure Northern blot showing the *IME2* transcript distribution on different polysome fractions.

RNA from different fractions was precipitated using isopropanol and resuspended in equal volumes of water. Equal volumes of RNA samples from each fraction were loaded on a formaldehyde agarose gel (lane 1: ssRNA Ladder, NEB), and after separation the RNA was transferred on to a nylon membrane, and hybridised to a P^{32} labelled *IME2* probe using standard protocols

(Primer sequences for IME2 probe: For_primerCTATCGCAGATACTGGCTGG; Rev_primer GTAGTAGATCCAACGATGAAC).
